# *Sinorhizobium meliloti* GAU-93 Inoculation Is Associated with Reduced Drought-Induced Oxidative Damage and Enhanced Flavonoid Accumulation and Antioxidant Capacity in Alfalfa

**DOI:** 10.3390/antiox15081040

**Published:** 2026-08-21

**Authors:** Xiaohu Wang, Jianhong Li, Xuelian Cui, Long Chen, Changning Li, Shuai Wang, Tuo Yao

**Affiliations:** 1 Pratacultural College, Gansu Agricultural University, Lanzhou 730070, China; 1073324020028@st.gsau.edu.cn (X.W.); cuixl@gsau.edu.cn (X.C.); 1073324120061@st.gsau.edu.cn (S.W.); 2 School of Biological Sciences and Technology, Liupanshui Normal University, Liupanshui 553004, China; lijianhong123@126.com; 3 Lanzhou Honggu District Science and Education Promoting Agriculture Demonstration Park Management Center, Lanzhou 730084, China; huangpp@st.gsau.edu.cn

**Keywords:** alfalfa, *Sinorhizobium meliloti* GAU-93, antioxidant, flavonoids, drought stress

## Abstract

This study investigated the effects of *Sinorhizobium meliloti* GAU-93 inoculation on the growth and drought responses of alfalfa (*Medicago sativa* L.). Alfalfa seeds were inoculated with the GAU-93 strain *Sinorhizobium meliloti* and subjected to four soil water content levels: 15% (severe drought), 30% (moderate drought), 45% (well-watered control), and 60% (high-moisture condition). Various growth parameters, antioxidant enzyme activities, flavonoid-related traits, and membrane lipid peroxidation were measured. Increasing drought severity reduced plant growth, fresh and dry biomass, and relative water content (RWC). GAU-93 inoculation was associated with smaller reductions in these traits across several water regimes. Inoculated plants also showed higher flavonoid contents and higher expression of several flavonoid biosynthetic genes, including *CHS*, *DFR*, *F3H*, and *PAL*. In addition, GAU-93 was associated with lower membrane lipid peroxidation and higher antioxidant enzyme activities. Overall, these findings suggest that *Sinorhizobium meliloti* GAU-93 may help alleviate drought-induced oxidative stress and support alfalfa growth under the tested water regimes. This study provides a basis for further evaluation of GAU-93 as a microbial strategy to improve drought tolerance in forage crops.

## 1. Introduction

Drought is a major environmental constraint that limits plant growth and development and contributes substantially to declines in crop yield and quality [1]. In arid regions, crop production depends largely on natural precipitation. However, even in irrigated systems, plants may still suffer from water deficit when irrigation is delayed or insufficient [2]. Over long-term evolutionary adaptation to water deficit, plants have developed multiple mechanisms and strategies to cope with drought stress. Plant responses to drought are generally classified into three principal strategies: drought escape, drought avoidance, and drought tolerance [3]. At the physiological level, plants enhance drought adaptation through modifications in root architecture, improved water-use efficiency, reduced water loss, metabolic adjustment, dormancy, and beneficial symbiotic interactions [4]. At the biochemical level, drought tolerance is supported by antioxidant defense, osmotic adjustment, and stress-related signal transduction, which together alleviate oxidative and cellular damage [5]. Accordingly, antioxidant enzyme activities, osmolyte accumulation, and secondary metabolite levels are widely used as indicators for evaluating plant responses to drought stress [6]. When plants are exposed to drought, increased levels of reactive oxygen species (ROS) are usually a response mechanism for plants to cope with adversity [7]. Although excessive ROS can lead to oxidative damage, moderate ROS production also plays an important role in regulating drought response in plants. Plants can strengthen their tolerance to drought by activating antioxidant defenses, particularly ROS-scavenging enzymes such as superoxide dismutase (SOD), ascorbate peroxidase (APX), catalase (CAT), and peroxidase (POD), thereby limiting drought-induced oxidative damage [8].

The plant antioxidant system and flavonoid metabolism are closely related through coordinated physiological and biochemical responses [9]. Flavonoids are important non-enzymatic antioxidants that work alongside enzymes such as SOD, CAT, and POD [10]. Under oxidative stress caused by factors such as UV exposure, pathogen attack, or drought, ROS can accumulate and may activate transcriptional responses that affect phenylpropanoid and flavonoid biosynthetic pathways [11]. Flavonoids can then contribute to ROS scavenging, metal ion chelation, and the maintenance of cellular redox balance [12].

Flavonoids are plant-derived polyphenolic secondary metabolites with diverse biological activities, including antioxidant, anti-inflammatory, and antiviral effects [13]. Their biological functions vary according to molecular structure and bioavailability. These compounds occur widely in fruits, leaves, stems, flowers, seeds, and nuts, where they contribute to multiple physiological and ecological processes [14]. In addition to determining the pigmentation of flowers, fruits, and leaves, flavonoids also participate in plant protection against environmental stress. For example, enhanced accumulation of kaempferol and quercetin increased non-enzymatic antioxidant capacity and restricted reactive oxygen species accumulation in rice exposed to drought and UV radiation [15]. In legumes, flavonoids released from roots also function as rhizosphere signals by binding to the rhizobial NodD protein and activating nod gene expression, thereby promoting the establishment of symbiotic nitrogen fixation [16].

For the legume-rhizobia symbiosis, drought poses a dual threat. It directly impairs plant physiological processes, such as carbon metabolism and energy provision, which are essential for fueling nitrogen fixation [17]. Simultaneously, it can disrupt the symbiotic relationship—from the initial infection stages through nodule development and function—by altering rhizobial survival, reducing nodule number and mass, and impairing the activity of the nitrogenase enzyme within the nodule [17]. Previous studies on maize, alfalfa, and wheat have highlighted the important roles of beneficial rhizosphere microorganisms in alleviating the adverse effects of drought stress [18,19,20,21]. Alfalfa inoculated with engineered Sinorhizobium meliloti strains showed less severe wilting and chlorosis and maintained a higher relative water content under drought stress [20]. Similarly, inoculation of maize with Rhizophagus intraradices promoted plant growth, phosphorus accumulation, fresh biomass, and water-use efficiency, while reducing plant C and N ratios under water-deficit conditions [19]. These findings indicate that beneficial microorganisms can improve plant drought adaptation by supporting water status, nutrient acquisition, and biomass production.

Alfalfa (*Medicago sativa* L.) is one of the most important perennial forage legumes worldwide and is extensively cultivated in arid and semi-arid regions, where drought stress represents a major environmental constraint limiting forage productivity. Although the detrimental effects of drought on alfalfa growth and rhizobial symbiosis have been widely documented, the physiological and molecular mechanisms underlying rhizobium-mediated drought tolerance remain largely unresolved. In particular, the extent to which rhizobial inoculation modulates flavonoid biosynthesis and antioxidant defense pathways to alleviate drought-induced oxidative stress has received limited attention.

To address this knowledge gap, the present study investigated the effects of *Sinorhizobium meliloti* GAU-93 on the growth and drought tolerance of alfalfa under different soil water regimes. GAU-93 was selected as the inoculant in this study because it is an alfalfa-associated rhizobial strain with potential relevance for improving symbiotic performance under water-limited conditions. Although several *S. meliloti strains* have been reported to affect alfalfa growth and stress responses, the role of GAU-93 in regulating alfalfa physiological responses under different soil water availability levels has not been fully characterized. Therefore, we comprehensively evaluated plant growth characteristics, relative water status, antioxidant enzyme activities, membrane lipid peroxidation, flavonoid accumulation, and the expression patterns of key genes involved in flavonoid biosynthesis. By integrating physiological and molecular analyses, this study aimed to elucidate whether GAU-93 enhances drought adaptation through the coordinated regulation of flavonoid metabolism and antioxidant defense systems. The findings provide novel insights into the mechanisms by which beneficial rhizobia confer drought resilience and offer a promising biological strategy for improving alfalfa productivity and sustainability under water-limited conditions.

## 2. Materials and Methods

### 2.1. Materials

Alfalfa (*Medicago sativa*) cultivar ‘Gannong No. 5’ seeds were provided by the College of Pratacultural Science, Gansu Agricultural University.

The bacterial strain used in this study was provided by the Grassland Microorganisms Research Group from the College of Pratacultural Science, Gansu Agricultural University, China, and was reported as non-pathogenic. The bacterial strain used in this study was *S. meliloti* GAU-93, which was identified by 16S rRNA gene sequence analysis and preserved in our laboratory. The 16S rRNA gene sequence showed high similarity to *S. meliloti*, confirming its taxonomic identity. Therefore, GAU-93 was used as the rhizobial inoculant for alfalfa in this study [20].

### 2.2. Methods

Alfalfa seeds were surface-sterilized with 1% (*v*/*v*) sodium hypochlorite solution for 10 min, followed by three rinses with sterile distilled water. After air-drying in the shade, plump seeds were selected for planting. Seeds were sown in autoclaved substrate (peat:vermiculite:perlite = 3:1:1; pH 6.8–7.0) in plastic pots (9 cm × 12 cm × 13 cm) and grown in a controlled chamber (16/8 h light/dark, 25 ± 1 °C/22 ± 1 °C day/night, 65–75% RH, 500–700 μmol m^−2^ s^−1^). Five seeds were initially sown in each pot and then thinned to three uniform seedlings at the true leaf stage. The experiment followed a factorial design with two factors: inoculation (Sinorhizobium meliloti GAU-93 vs. sterile medium) and soil water content (SWC), giving eight treatment combinations. Each biological replicate corresponded to one pot, with four pots per treatment; the three plants within each pot were maintained as within-pot subsamples and pooled when destructive biochemical or molecular measurements were performed. Sinorhizobium meliloti GAU-93 was cultured in yeast extract mannitol broth (YMB) at 28 °C with shaking at 180 rpm until the logarithmic growth phase. The bacterial culture was centrifuged at 6000× *g* for 10 min, and the cell pellet was washed twice with sterile distilled water. GAU-93 was activated in sterile liquid yeast extract mannitol medium and cultured to the logarithmic phase before inoculation; the suspension was adjusted to approximately 1 × 10^8^ CFU mL^−1^, and 2 mL of the suspension was applied to the root zone of each inoculated pot. Non-inoculated controls received the same volume of sterile medium. Successful inoculation was checked phenotypically by nodule formation at harvest. Plants were supplied with nitrogen-deficient Holgan nutrient solution every three days. After establishment, all pots were maintained at 45% SWC until the late tillering stage, when water treatments were initiated. SWC levels were established gravimetrically. Briefly, the water-holding capacity of the substrate was determined from the mass difference between the saturated, freely drained substrate and oven-dried substrate. Target pot mass was then calculated for 15%, 30%, 45%, or 60% SWC, and water loss was replenished daily by weighing each pot and adding sterile water to restore the target mass. Specifically, 15% and 30% SWC were treated as drought stress conditions, 45% SWC as the control condition, and 60% SWC as a high-moisture non-stress treatment. Treatments were maintained until the early flowering stage, when leaves and root nodules were harvested for analysis. All samples were collected at the end of the drought treatment between 08:00 and 10:00 a.m. to avoid possible effects of diurnal variation.

### 2.3. Biological SEM Microscopy

After harvesting, the roots were carefully washed with distilled water to remove adhering soil. Visible nodules attached to the root system were counted manually for each plant. Nodule number was expressed as the number of nodules per plant, and nodule fresh weight was measured immediately after counting. Freshly harvested root nodules from the treatment group were dissected into approximately 1 mm^3^ tissue blocks. Alternatively, samples were collected to yield approximately 10^7^ cells, with pellet volumes ranging between that of a grain of rice and a soybean. The samples were immediately transferred to 1.5 mL or 2 mL centrifuge tubes containing 2.5% glutaraldehyde and fixed at 4 °C for 12–24 h.

For scanning electron microscopy, the fixative was removed, and the samples were washed three times with 0.1 M phosphate buffer (PB, pH 7.0) for 15 min each. The tissues were then post-fixed in 1% osmium tetroxide for 1-2 h, followed by three additional washes with 0.1 M PB (pH 7.4), each lasting 15 min. Samples were dehydrated in a graded ethanol series of 30%, 50%, 70%, 80%, 90%, and 95% for 15 min at each concentration, followed by two 20 min washes in absolute ethanol. After critical-point drying, the samples were mounted on specimen stubs using conductive carbon adhesive and sputter-coated with platinum for approximately 120 s. The prepared specimens were examined using a scanning electron microscope (Regulus 8100, Hitachi, Japan).

### 2.4. Leaf Relative Water Content (RWC), Height, Plant Dry and Fresh Weight

Alfalfa leaves were collected from the same position on each plant, rinsed twice with deionized water, gently blotted dry with absorbent paper, and immediately weighed to determine fresh weight. The samples were then placed in paper envelopes, heated at 105 °C for 15 min to halt metabolic activity, and subsequently dried at 70 °C to constant weight. The final dry weight was then recorded.

The RWC of fully expanded leaves was determined according to [(FW − DW)/(TW − DW)] × 100, where FW, DW, and TW are leaf fresh weight, dry weight, and saturated fresh weight, respectively [22,23]. For TW determination, fully expanded leaves were immersed in distilled water in the dark at 4 °C until full rehydration, gently blotted dry with absorbent paper, and weighed immediately.

### 2.5. Measurement of Physiological Parameters

The activities of four key antioxidant enzymes were determined using established spectrophotometric methods and commercial assay kits according to the manufacturers’ instructions. SOD activity was measured using nitroblue tetrazolium (NBT) colorimetry according to Abogadallah et al. [24], based on inhibition of NBT photoreduction at 560 nm. CAT activity was determined by monitoring H_2_O_2_ decomposition as the decrease in absorbance at 240 nm [25]. POD activity was determined using guaiacol as the hydrogen donor and monitoring tetraguaiacol formation at 470 nm [26]. APX activity was determined using the ascorbate oxidation method by monitoring the decrease in absorbance at 290 nm, which is the conventional wavelength for APX assays. The contents of H_2_O_2_, MDA, and O_2_•^−^ were determined using the Hydrogen Peroxide Content Assay Kit, Malondialdehyde Content Assay Kit, and Superoxide Anion Content Assay Kit, respectively, according to the manufacturer’s instructions (Solarbio Science & Technology Co., Ltd., Beijing, China) [27]. Fresh samples (0.2 g) were homogenized with 5 mL of distilled water and extracted in a boiling water bath for 30 min. After cooling, the homogenate was centrifuged at 12,000× *g* for 10 min, and the supernatant was collected for soluble sugar determination. Soluble sugar content was quantified using the anthrone colorimetric method [28]. Briefly, an aliquot of the extract was reacted with anthrone reagent, and the absorbance was measured at 620 nm. Glucose was used to generate the standard curve, and soluble sugar content was calculated according to the standard curve and expressed as mg g^−1^ fresh weight.

Soluble protein content was determined using the Coomassie Brilliant Blue G-250 method as described in [29], with minor modifications. Approximately 0.2 g of fresh tissue was ground and extracted with 5 mL of distilled water. The homogenate was centrifuged at 12,000× *g* for 20 min at 4 °C, and the supernatant was collected for analysis. Subsequently, 1 mL of the supernatant was mixed with 5 mL of Coomassie Brilliant Blue reagent. After reacting for 2 min at room temperature, the absorbance was measured at 595 nm. Bovine serum albumin (BSA) was used as the standard to generate a calibration curve. The soluble protein concentration in each extract was calculated from the standard curve, and the final soluble protein content was expressed as mg g^−1^ fresh weight (FW) according to the following formula: soluble protein content = C × V × D/m, where C is the protein concentration calculated from the standard curve (mg mL^−1^), V is the extraction volume (mL), D is the dilution factor, and m is the fresh sample weight (g).

### 2.6. Histochemical Staining Assay

Histochemical staining was used for qualitative visualization of H_2_O_2_ and O_2_•^−^ accumulation. For DAB staining, alfalfa leaves were immersed in 1 mg mL^−1^ 3,3’-diaminobenzidine (DAB) solution prepared in sterile distilled water; the solution pH was adjusted to approximately 3.8 before use. For NBT staining, leaves were immersed in NBT solution prepared with HEPES buffer (2.38 g HEPES and 0.49 g NBT in 100 mL sterile distilled water). Samples were vacuum-infiltrated briefly to improve reagent penetration and then incubated at room temperature in the dark for 24 h. After staining, chlorophyll was removed by incubating leaves in 80% (*v*/*v*) ethanol at approximately 80 °C until the background became clear. Stained leaves were photographed under identical imaging conditions. The staining assay was interpreted qualitatively; darker brown DAB staining indicated greater H_2_O_2_ accumulation, whereas darker blue NBT staining indicated greater O_2_•^−^ accumulation.

### 2.7. Determination of Flavonoid Content

The concentrations of daidzein and genistein were determined by high-performance liquid chromatography (HPLC) [30]. The samples used for this analysis were dry plant materials. Fresh plant tissues were first collected and gently rinsed with distilled water to remove surface impurities. The samples were then blotted dry with filter paper and oven-dried at 105 °C for 30 min to stop enzymatic activity, followed by drying at 65 °C until a constant weight was achieved. The dried samples were ground into a fine powder using a grinder and passed through a 1 mm sieve. The powdered samples were stored in sealed bags at room temperature in a dry environment until further analysis. A powdered sample (1.0 g) was extracted with chromatographic-grade methanol at a material-to-solvent ratio of 1:20 (g:mL) for 4 h, followed by ultrasonic extraction for 30 min. The residue was extracted a second time with 20 mL methanol for 30 min. The two extracts were combined, concentrated under vacuum at 30 °C, and reconstituted to 10 mL with methanol. Before HPLC analysis, extracts were filtered through a 0.22 μm PTFE membrane. Chromatographic separation was performed on a reversed-phase C18 column using a methanol-water mobile phase, with UV detection at 260 nm, a flow rate of 1.0 mL min^−1^, and a column temperature of 30 °C. Daidzein and genistein were identified by comparing retention times with authentic standards and quantified using external standard calibration curves.

### 2.8. CHI and CHS Activities Determination

For CHS and CHI enzyme extraction, 100 mg of fresh plant tissue was homogenized in 1 mL of pre-cooled extraction buffer and centrifuged at 10,000× *g* for 15 min at 4 °C. The supernatant was collected immediately and used as the crude enzyme extract. Enzyme activity was calculated from the absorbance change relative to reaction time and sample fresh weight; one unit of enzyme activity was defined as the amount of enzyme causing a change of 0.01 absorbance units per minute under the assay conditions.

CHS Activity Assay: The reaction was initiated by mixing 100 μL of the enzyme extract with 1 mL of reaction buffer (10 mM MgCl_2_, 50 μM naringenin chalcone). After incubation at 30 °C for 30 min, the reaction was terminated by adding 200 μL of 1 M HCl. Absorbance was measured at 390 nm.

CHI Activity Assay: similarly, 100 μL of the enzyme extract was combined with 1 mL of reaction buffer (10 mM MgCl_2_, 50 μM chalcone substrate). After a 30 min incubation at 30 °C, the reaction was stopped by adding 200 μL of 1 M HCl. Absorbance was recorded at 310 nm. Standard curves prepared with the corresponding flavonoid standards were used when converting absorbance to product concentration.

### 2.9. Quantitative Real-Time PCR (qRT-PCR)

#### 2.9.1. Extraction of Total RNA

Total root RNA (1 μg) was reverse-transcribed using the FastQuant First Strand cDNA Synthesis Kit (TIANGEN, Beijing, China) following the manufacturer’s instructions.

#### 2.9.2. cDNA Chain Synthesis

The extracted RNA was reverse-transcribed into cDNA according to the kit protocol, with incubation at 37 °C for 15 min followed by 85 °C for 5 s and storage at 4 °C.

#### 2.9.3. qRT-PCR

Quantitative real-time PCR (qRT-PCR) was performed using an ABI StepOnePlus Real-Time PCR System (Applied Biosystems, Carlsbad, CA, USA) according to the manufacturer’s instructions. The relative expression levels of the nodulation-related genes *NOD1* and *NOD2* and the flavonoid biosynthesis-related genes *PAL*, *CHS*, *F3H*, and *DFR* were determined. MsActin was used as the internal reference gene, following Gao et al. (2024) [29]. Gene-specific primers were designed based on the corresponding coding sequences, and the primer sequences are listed in Table S1. Relative gene expression was calculated using the 2^−ΔΔ*C*t^ method [31].

### 2.10. Data Statistics and Analysis

All physiological and biochemical data were compiled using Microsoft Excel 2016 (Microsoft Corporation, Redmond, WA, USA). Data are presented as mean ± standard error (SE), with four biological replicates for each treatment. Data were analyzed using two-way analysis of variance (ANOVA) to evaluate the effects of inoculation, soil water content (SWC), and their interaction. When significant main or interaction effects were detected, post hoc comparisons were performed using Tukey-adjusted multiple comparisons or simple-effects comparisons between inoculated and non-inoculated plants within each SWC level, as appropriate. The assumptions of normality and homogeneity of variance were assessed using residual diagnostics and Levene’s test, respectively. Statistical significance was determined at *p* < 0.05. Graphs and visualizations were generated using OriginPro 2021 (OriginLab Corporation, Northampton, MA, USA).

## 3. Results

### 3.1. Scanning Electron Microscopy Ultrastructural Observation of Alfalfa Root Nodules

**Figure 1:**
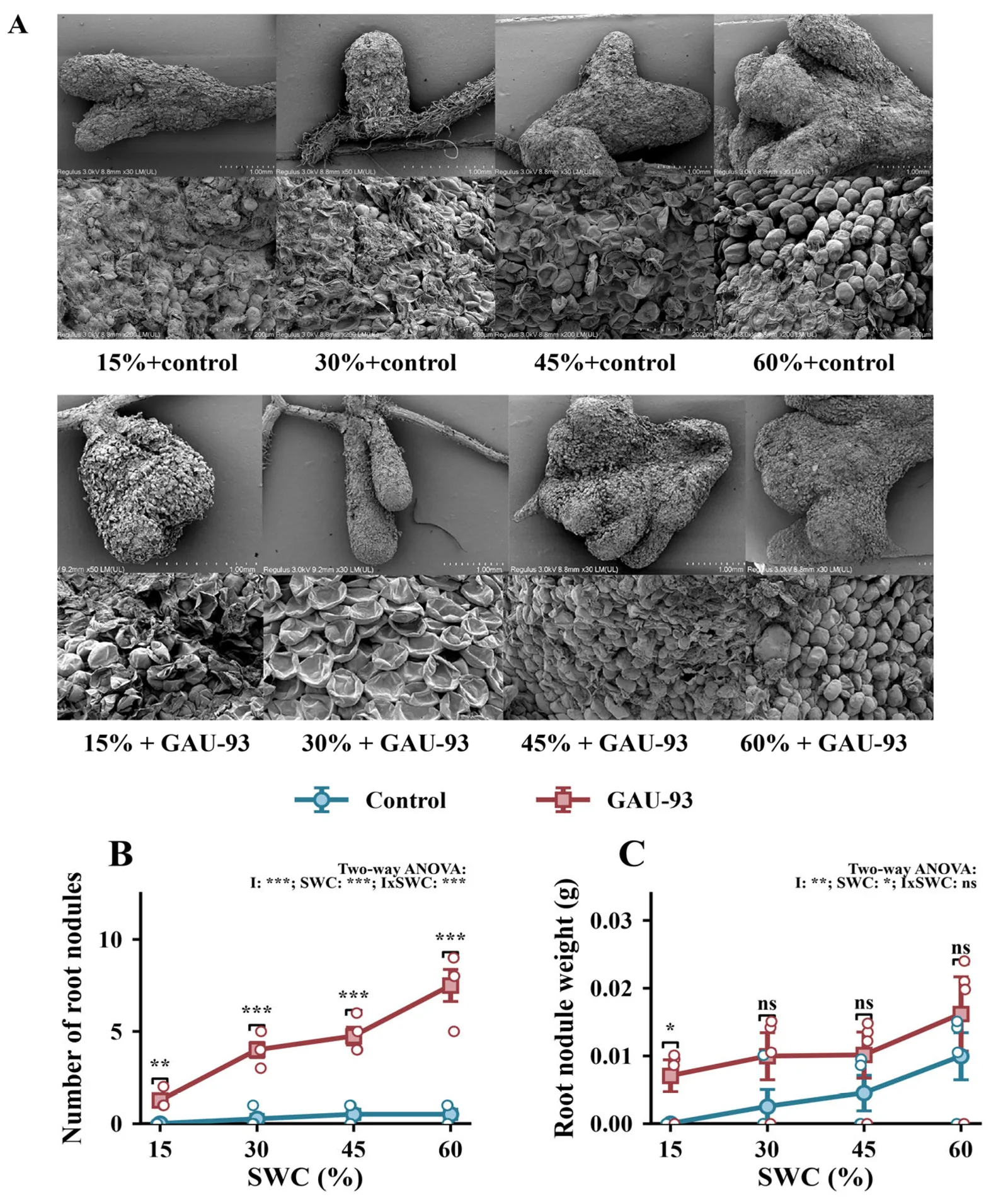
Effects of control and GAU-93 on alfalfa root nodule morphology (**A**), nodule number (**B**), and nodule weight (**C**) under different SWC levels. Data are shown as mean ± SE, n = 4. Two-way ANOVA was used to test the effects of inoculation, SWC, and their interaction. Asterisks indicate Tukey-adjusted simple-effect comparisons between inoculated and non-inoculated plants within the same SWC level: * *p* < 0.05, ** *p* < 0.01, *** *p* < 0.001, ns, not significant.

As shown in Figure 1, root nodule morphology, nodule number, and nodule weight varied across SWC levels and inoculation treatments. SEM observations revealed visible differences in nodule surface morphology and structural organization among treatments. Two-way ANOVA showed significant effects of inoculation and SWC and their interaction on the number of root nodules. Compared with uninoculated controls, GAU-93-inoculated plants had significantly higher nodule numbers at all SWC levels, with the highest values observed under 60% SWC. For root nodule weight, two-way ANOVA showed significant effects of inoculation and SWC, whereas the inoculation × SWC interaction was not significant. Pairwise comparisons showed that GAU-93 significantly increased nodule weight only at 15% SWC, while differences at 30%, 45%, and 60% SWC were not significant. GAU-93-inoculated plants had significantly higher nodule numbers than uninoculated plants at all SWC levels, whereas a significant increase in nodule weight was observed only at 15% SWC.

### 3.2. Effect of GAU-93 on Plant Dry and Fresh Weight and Leaf Relative Water Content (RWC) in Alfalfa Under Drought Stress

**Figure 2:**
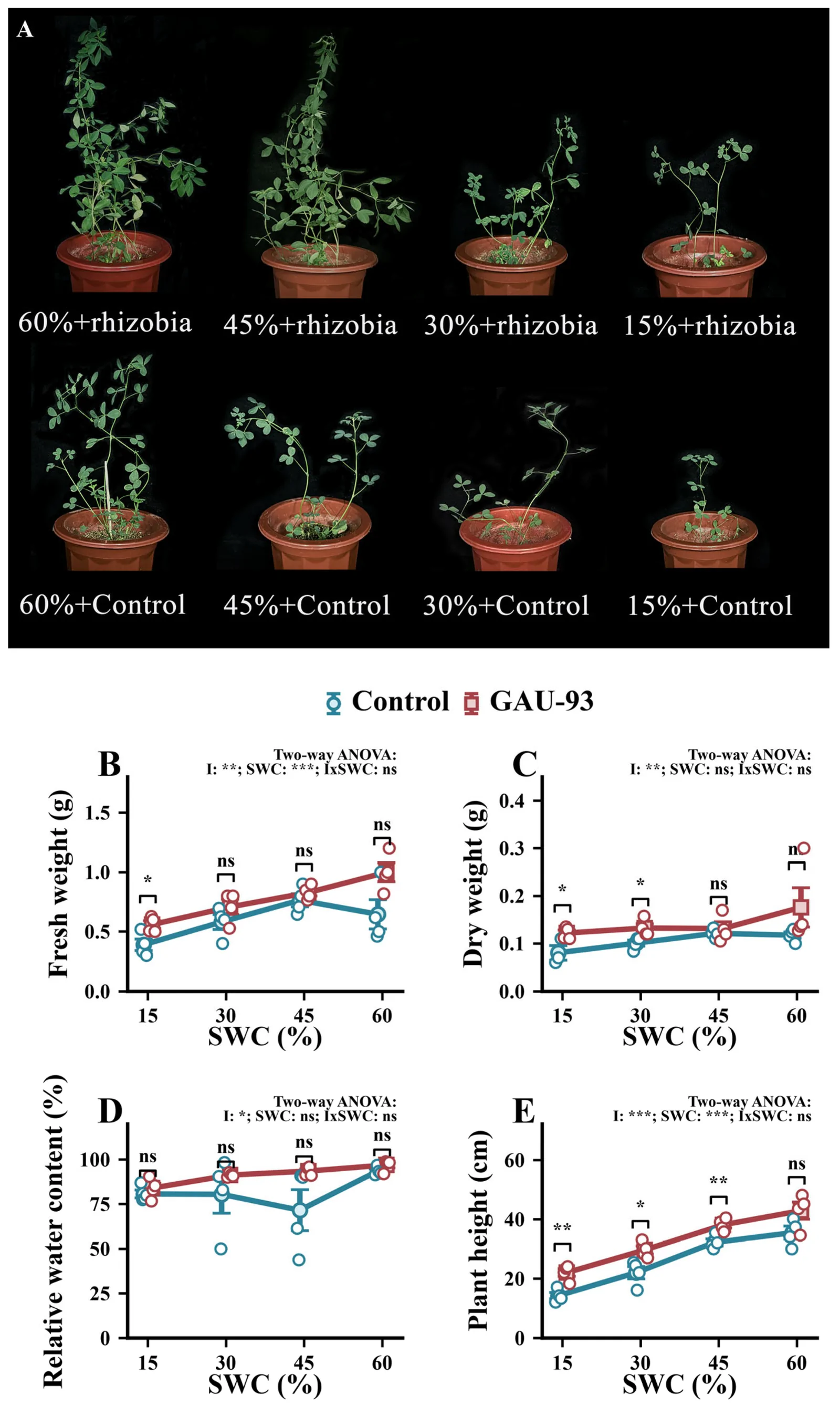
Effects of GAU-93 on alfalfa under different SWC levels. (**A**), Representative phenotypes of alfalfa plants under different treatments. (**B**), leaf dry weight (**C**), plant height (**D**), and relative water content (RWC; **E**) in alfalfa under different SWC levels. Data are shown as mean ± SE, n = 4. Two-way ANOVA was used to test the effects of inoculation, SWC, and their interaction. Asterisks indicate Tukey-adjusted simple-effect comparisons between inoculated and non-inoculated plants within the same SWC level: * *p* < 0.05, ** *p* < 0.01, *** *p* < 0.001, ns, not significant.

As shown in Figure 2, plant growth traits varied across SWC levels and inoculation treatments. Phenotypic observations showed that plant growth was reduced under lower SWC levels, particularly at 15% and 30% SWC, whereas plants under 45% and 60% SWC generally maintained better growth status. Two-way ANOVA showed that inoculation significantly affected fresh weight, dry weight, relative water content, and plant height, while SWC significantly affected fresh weight and plant height. The inoculation × SWC interaction was not significant for these traits. Compared with uninoculated controls under the same SWC level, GAU-93-inoculated plants showed significantly higher fresh weight only at 15% SWC. Dry weight was significantly higher in GAU-93-inoculated plants at 15% and 30% SWC, but not at 45% or 60% SWC. Although the main effect of inoculation on relative water content was significant, pairwise comparisons between inoculated and uninoculated plants were not significant at individual SWC levels. For plant height, GAU-93-inoculated plants showed significantly greater values at 15%, 30%, and 45% SWC, whereas the difference at 60% SWC was not significant. Overall, these results indicate that GAU-93 inoculation was associated with changes in several growth-related traits, but the magnitude and significance of the response depended on the measured trait and SWC level.

### 3.3. Effect of GAU-93 on Permeable Substance Content in Alfalfa Under Drought Stress

**Figure 3:**
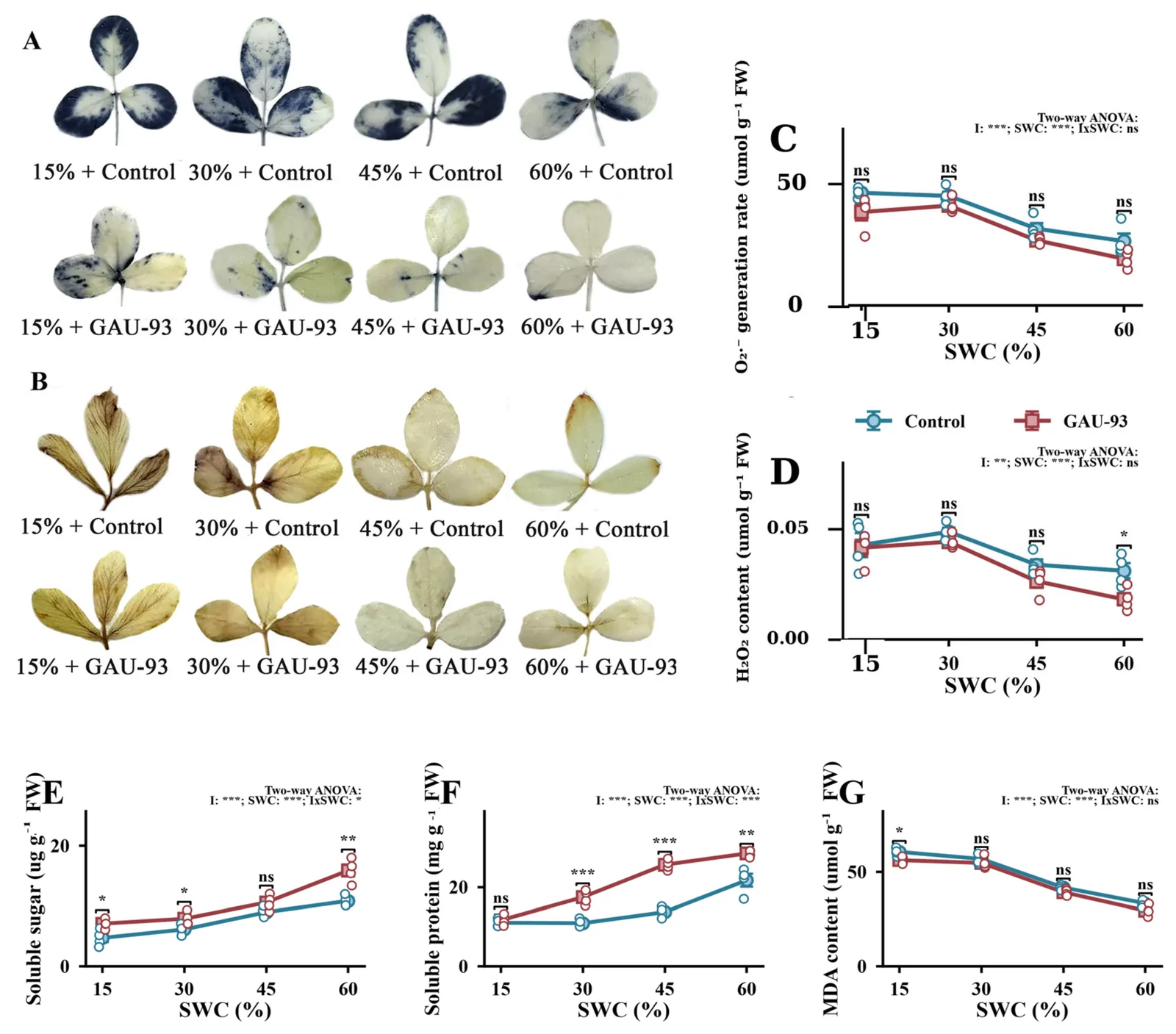
Effects of GAU-93 and control inoculation on ROS accumulation and osmolyte-related traits in alfalfa leaves under different SWC levels. (**A**) NBT staining for O_2_•^−^; (**B**) DAB staining for H_2_O_2_; (**C**) H_2_O_2_ content; (**D**) O_2_•^−^ content; (**E**) soluble sugar; (**F**) soluble protein; and (**G**) MDA content. Data are shown as mean ± SE, n = 4. Two-way ANOVA was used to test the effects of inoculation, SWC, and their interaction. Asterisks indicate Tukey-adjusted simple-effect comparisons between inoculated and non-inoculated plants within the same SWC level: * *p* < 0.05, ** *p* < 0.01, *** *p* < 0.001, ns, not significant.

As shown in Figure 3, ROS accumulation, osmotic adjustment-related traits, and lipid peroxidation varied across SWC levels and inoculation treatments. NBT and DAB staining showed stronger staining intensity under lower SWC levels, particularly in uninoculated plants, whereas GAU-93-inoculated plants generally displayed weaker staining under the corresponding SWC conditions. Consistently, biochemical assays showed that H_2_O_2_ content and O_2_•^−^ generation rate were affected by both SWC and inoculation, although pairwise differences between inoculated and uninoculated plants were limited at individual SWC levels. In the NBT staining assay, blue/purple staining indicates O_2_•^−^ accumulation, while in the DAB staining assay, brown staining indicates H_2_O_2_ accumulation. For H_2_O_2_ content, a significant difference between inoculated and uninoculated plants was observed at 60% SWC, while differences at 15%, 30%, and 45% SWC were not significant. O_2_•^−^ generation rate tended to be lower in GAU-93-inoculated plants than in controls, but no significant pairwise differences were detected at each SWC level.

Soluble sugar and soluble protein contents also differed among SWC and inoculation treatments. GAU-93-inoculated plants showed significantly higher soluble sugar content at 15%, 30%, and 60% SWC, whereas the difference at 45% SWC was not significant. Soluble protein content was significantly higher in GAU-93-inoculated plants at 30%, 45%, and 60% SWC, but not at 15% SWC. MDA content decreased with increasing SWC and was significantly lower in GAU-93-inoculated plants than in controls only at 15% SWC. Overall, these results indicate that GAU-93 inoculation was associated with changes in ROS-related indicators and osmotic adjustment traits, but the responses varied depending on the specific parameter and SWC level.

### 3.4. Effect of GAU-93 on Antioxidant Enzyme Activity in Alfalfa Under Drought Stress

**Figure 4:**
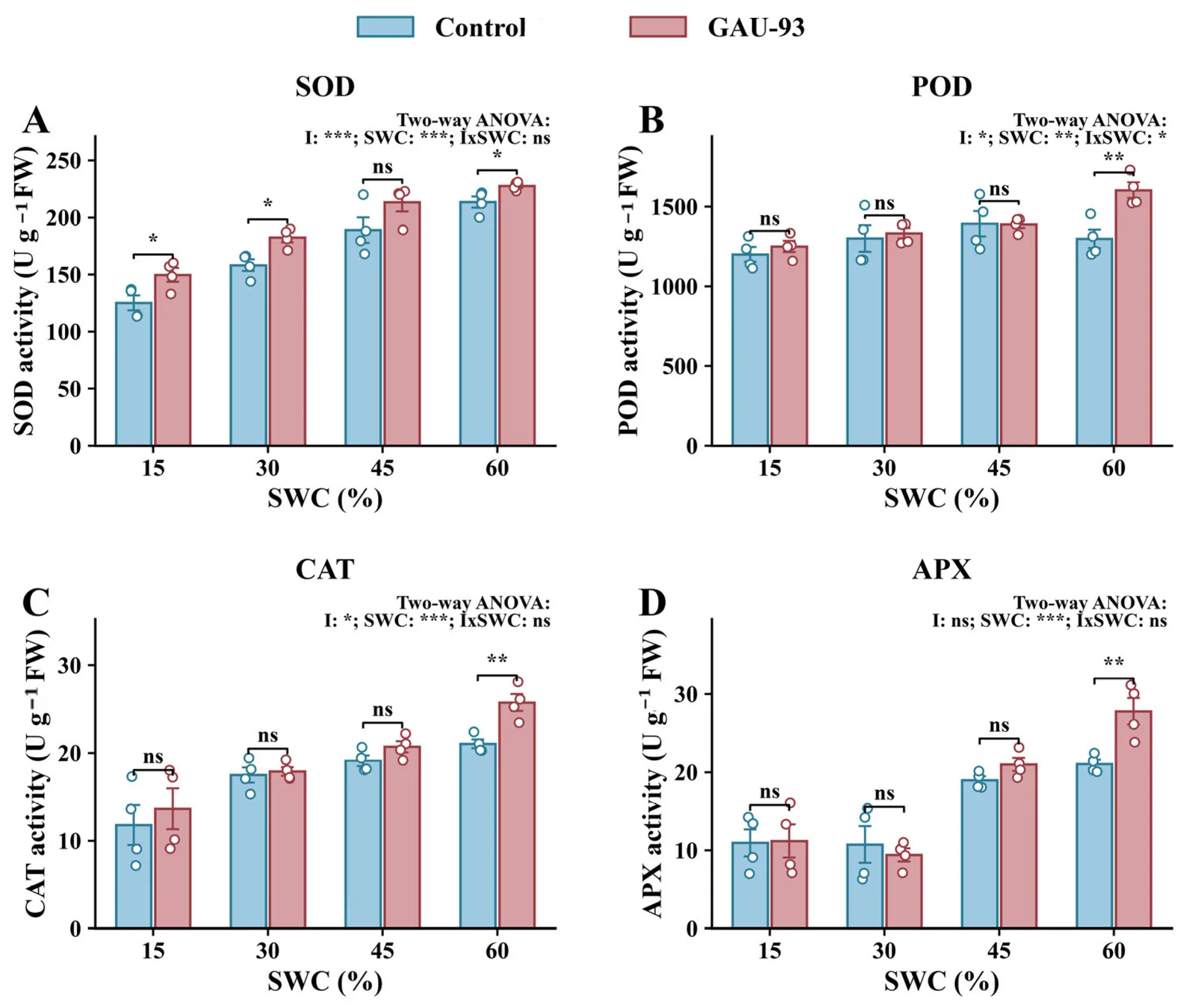
Effects of GAU-93 on CAT, APX, SOD, and POD activities in alfalfa under different SWC levels. (**A**) SOD activity; (**B**) POD activity; (**C**) CAT activity; (**D**) APX activity. Data are shown as mean ± SE, n = 4. Two-way ANOVA was used to test the effects of inoculation, SWC, and their interaction. Asterisks indicate Tukey-adjusted simple-effect comparisons between inoculated and non-inoculated plants within the same SWC level: * *p* < 0.05, ** *p* < 0.01, *** *p* < 0.001, ns, not significant.

As shown in Figure 4, the activities of antioxidant enzymes varied across SWC levels and inoculation treatments (Figure 4A–D). Two-way ANOVA showed that SWC significantly affected SOD, POD, CAT, and APX activities, indicating that antioxidant enzyme activities changed with soil water availability. Inoculation had significant effects on SOD, POD, and CAT activities, whereas its main effect on APX activity was not significant. For SOD, GAU-93-inoculated plants showed significantly higher activity than uninoculated controls at 15%, 30%, and 60% SWC, but not at 45% SWC. For POD, CAT, and APX, significant differences between inoculated and uninoculated plants were mainly observed at 60% SWC, while differences at 15%, 30%, and 45% SWC were generally not significant. Overall, these results indicate that antioxidant enzyme activities were influenced by soil water availability and, for some enzymes, by GAU-93 inoculation; however, the inoculation effect was enzyme- and SWC-dependent rather than consistent across all treatments.

### 3.5. Effect of GAU-93 on Flavonoid Content and Enzyme Activity in Alfalfa Under Drought Stress

**Figure 5:**
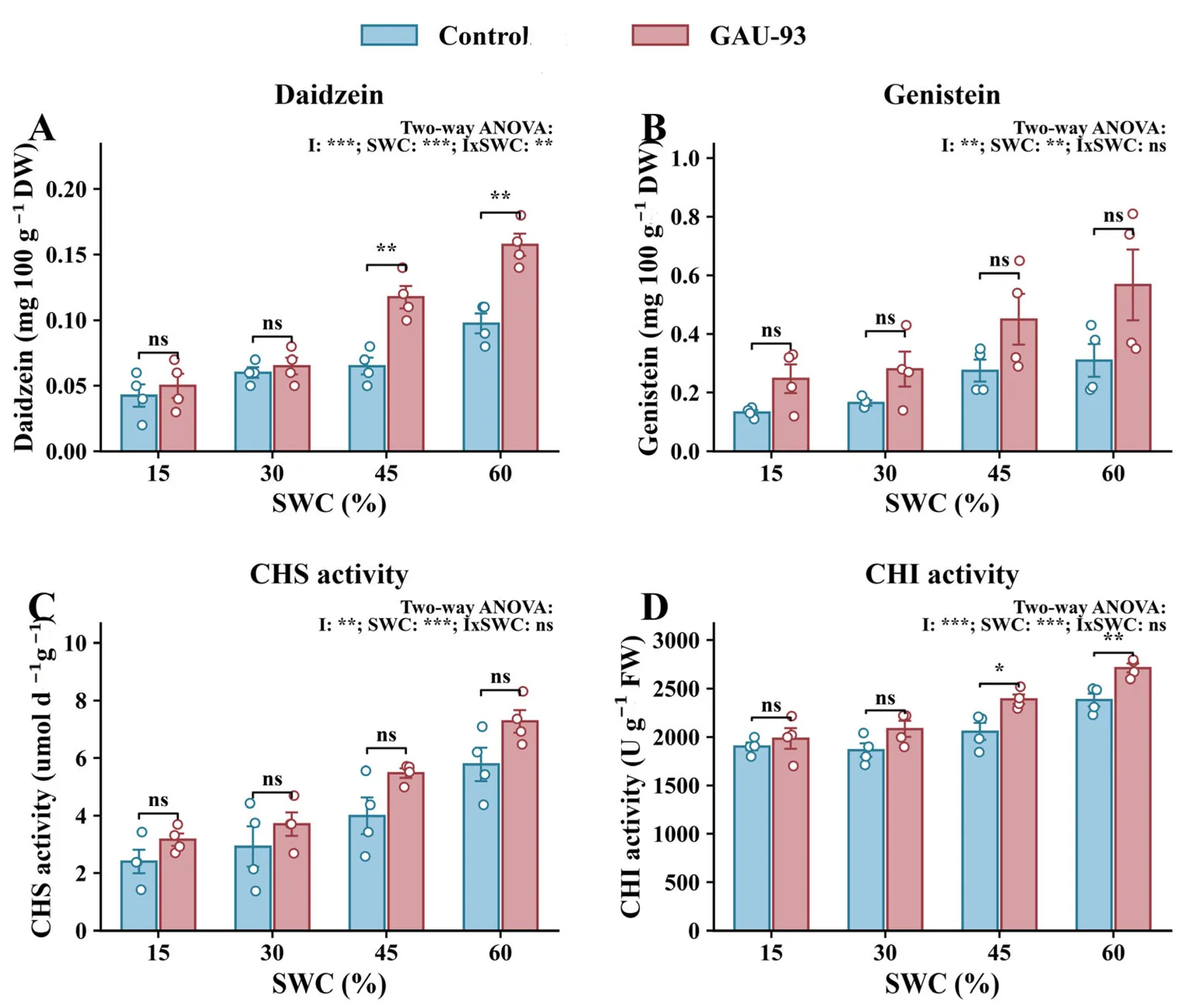
Effects of GAU-93 on daidzein content (**A**), genistein content (**B**), CHI activity (**C**), and CHS activity (**D**) in alfalfa under different SWC levels. Data are shown as mean ± SE, n = 4. Two-way ANOVA was used to test the effects of inoculation, SWC, and their interaction. Asterisks indicate Tukey-adjusted simple-effect comparisons between inoculated and non-inoculated plants within the same SWC level: * *p* < 0.05, ** *p* < 0.01, *** *p* < 0.001, ns, not significant.

As shown in Figure 5, daidzein, genistein, CHS activity, and CHI activity varied across different SWC levels and inoculation treatments (Figure 5A–D). Two-way ANOVA showed significant effects of inoculation and SWC on daidzein, genistein, CHS activity, and CHI activity, whereas the inoculation × SWC interaction was significant only for daidzein. For daidzein, GAU-93-inoculated plants showed significantly higher levels than uninoculated controls at 45% and 60% SWC, while no significant differences were observed at 15% and 30% SWC. Genistein content tended to be higher in GAU-93-inoculated plants across SWC levels, but the pairwise differences between inoculated and uninoculated plants were not significant at each individual SWC level. CHS activity also showed an increasing trend with increasing SWC, and GAU-93-inoculated plants generally had higher values than controls; however, no significant pairwise differences were detected under the same SWC level. For CHI activity, GAU-93 inoculation significantly increased enzyme activity at 45% and 60% SWC, whereas the differences at 15% and 30% SWC were not significant. GAU-93 inoculation was associated with changes in selected flavonoid-related metabolites and enzyme activities, although these responses varied among indicators and SWC levels; thus, the potential contribution of flavonoid metabolism to drought tolerance remains hypothetical and requires further experimental validation.

### 3.6. Effects of GAU-93 on the Expression Level of NOD-1, NOD-2, CHS, PAL, F3H and DFR Gene During Drought Stress

**Figure 6:**
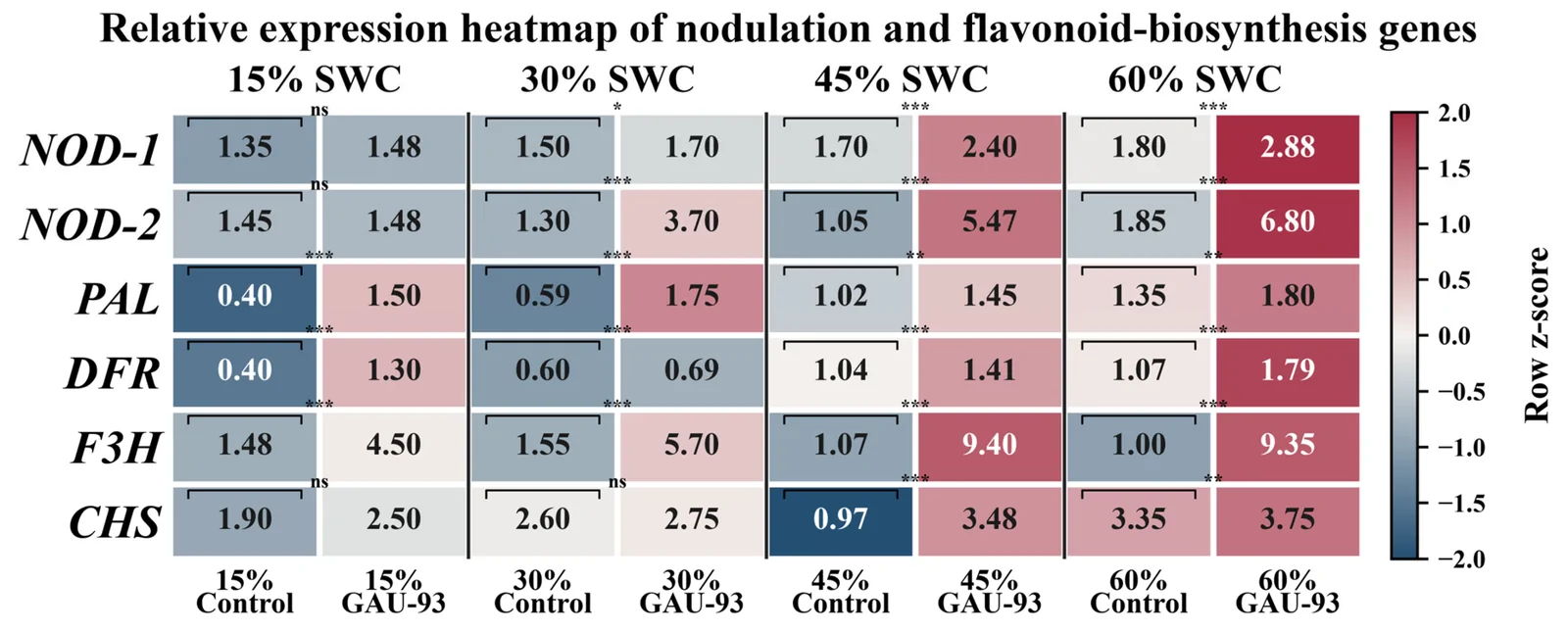
Relative expression heatmap of nodulation- and flavonoid-biosynthesis-related genes in alfalfa under different soil water content (SWC) levels with or without GAU-93 inoculation. Values shown in each cell represent the mean relative expression level (n = 4). The color scale indicates row-wise z-scores, with blue and red representing lower and higher relative expression within each gene, respectively. Control indicates non-inoculated plants. Asterisks indicate Tukey-adjusted simple-effect comparisons between inoculated and non-inoculated plants within the same SWC level: * *p* < 0.05, ** *p* < 0.01, *** *p* < 0.001, ns, not significant.

As shown in Figure 6, the relative expression levels of nodulation- and flavonoid biosynthesis-related genes varied across SWC levels and inoculation treatments. Compared with the corresponding uninoculated controls, GAU-93-inoculated plants generally showed higher transcript levels for several genes, although the response was gene- and treatment-specific rather than uniform. At 15% SWC, *PAL*, *DFR*, and *F3H* were significantly higher in GAU-93-inoculated plants, whereas *NOD-1*, *NOD-2*, and *CHS* showed no significant difference between inoculated and uninoculated plants. At 30% SWC, GAU-93 inoculation was associated with significant increases in *NOD-1*, *NOD-2*, *PAL*, *DFR*, and *F3H*, while *CHS* showed no significant change. Under 45% and 60% SWC, most examined genes showed significantly higher expression in GAU-93-inoculated plants than in the corresponding controls, with relatively strong responses observed for *NOD-2* and *F3H*. Overall, these results indicate that GAU-93 inoculation was associated with differential transcriptional responses of selected nodulation- and flavonoid-related genes under different soil water availability conditions.

## 4. Discussion

Drought stress affects plant growth and agricultural productivity and can also disrupt plant interactions with beneficial microorganisms such as rhizobia [32]. In this study, inoculation with Sinorhizobium meliloti GAU-93 was associated with smaller decreases in growth, relative water content (RWC), and several physiological indices under the tested SWC regimes. GAU-93 was also associated with lower membrane lipid peroxidation, higher antioxidant enzyme activities, and higher flavonoid accumulation and expression of several flavonoid biosynthetic genes. Notably, the inoculation × SWC interaction was not significant for most measured traits, indicating that GAU-93 did not induce a distinct drought-specific defense response. Instead, its effects were generally additive across water regimes, suggesting that inoculation may support stress tolerance primarily by maintaining plant physiological and metabolic functions under water-limited conditions.

The symbiotic nitrogen-fixing relationship between rhizobia and legumes enables nitrogen acquisition in exchange for plant-derived carbon and a suitable ecological niche for the bacteria [33]. However, drought can disrupt root water uptake and impair key morphological and physiological processes [34]. The root system and its interaction with rhizobia play an important role in coordinating plant growth, nutrient assimilation, and adaptation under water-limited conditions [35]. Consistent with previous reports, GAU-93 was associated with higher nodule number and nodule biomass, and with higher RWC, fresh weight, and dry weight at several SWC levels [36]. These patterns are more consistent with a general maintenance of plant water status and growth across the tested water regimes than with the activation of a specific drought-induced response.

In addition to its effects on growth, GAU-93 inoculation was associated with changes in antioxidant capacity under the tested water regimes. Under stress, plants regulate oxidative damage through enzymatic and non-enzymatic antioxidants [37]. SOD, CAT, APX, and POD are among the enzymes involved in ROS scavenging [38,39]. In this study, GAU-93 was associated with higher SOD, APX, CAT, and POD activities at several SWC levels, together with lower H_2_O_2_ and MDA levels in some treatments. Because significant inoculation × SWC interactions were generally absent, the observed differences in antioxidant traits should not be interpreted as evidence that GAU-93 induced a drought-specific antioxidant defense program. Rather, GAU-93 may help maintain antioxidant capacity and limit oxidative deterioration across different water regimes. Because the biochemical measurements were normalized to fresh weight, differences in tissue water content among the SWC treatments may have partially influenced the calculated concentrations. Therefore, these results should primarily be interpreted as relative changes among treatments rather than absolute changes in metabolite accumulation.

Beyond its effects on antioxidant enzymes, GAU-93 was also associated with higher flavonoid levels and higher expression of several flavonoid biosynthetic genes [40]. Drought can disrupt phenylpropanoid and flavonoid metabolism and alter the expression and activity of enzymes such as PAL, CHS, CHI, and IFS [41]. Genistein and daidzein are representative isoflavones in legumes and may have specific significance in plant adaptation to drought stress. Drought stress usually induces excessive ROS accumulation, leading to oxidative damage [42]. The accumulation of genistein and daidzein may contribute to the maintenance of ROS homeostasis and the reduction in oxidative injury. Moreover, these compounds are closely related to legume-rhizobium interactions and may be involved in nodulation-associated signaling. Thus, the increased levels of genistein and daidzein in GAU-93-inoculated plants indicate that GAU-93 may improve drought tolerance by modulating isoflavone metabolism [43]. However, further functional studies are required to confirm the direct roles of these compounds in alfalfa drought tolerance [44]. In this study, GAU-93-treated plants showed higher *CHS*, *CHI*, and *F3H* transcript levels than the corresponding controls at several SWC levels. Similarly, the changes in flavonoid-related metabolites, enzyme activities, and gene expression were not associated with a consistent inoculation × SWC interaction, suggesting that GAU-93 may help maintain secondary metabolic activity rather than induce a distinct drought-specific flavonoid response.

Finally, drought can alter nodulation-related gene expression by affecting symbiotic signaling between legumes and rhizobia [45]. Root-secreted flavonoids are important in rhizobial signaling and may influence nod gene activation and Nod factor synthesis [46,47]. In this study, nodulation-related and flavonoid-related genes showed treatment-dependent expression patterns across the SWC gradient, suggesting that rhizobial responses were influenced by water regime.

## 5. Conclusions

**Figure 7:**
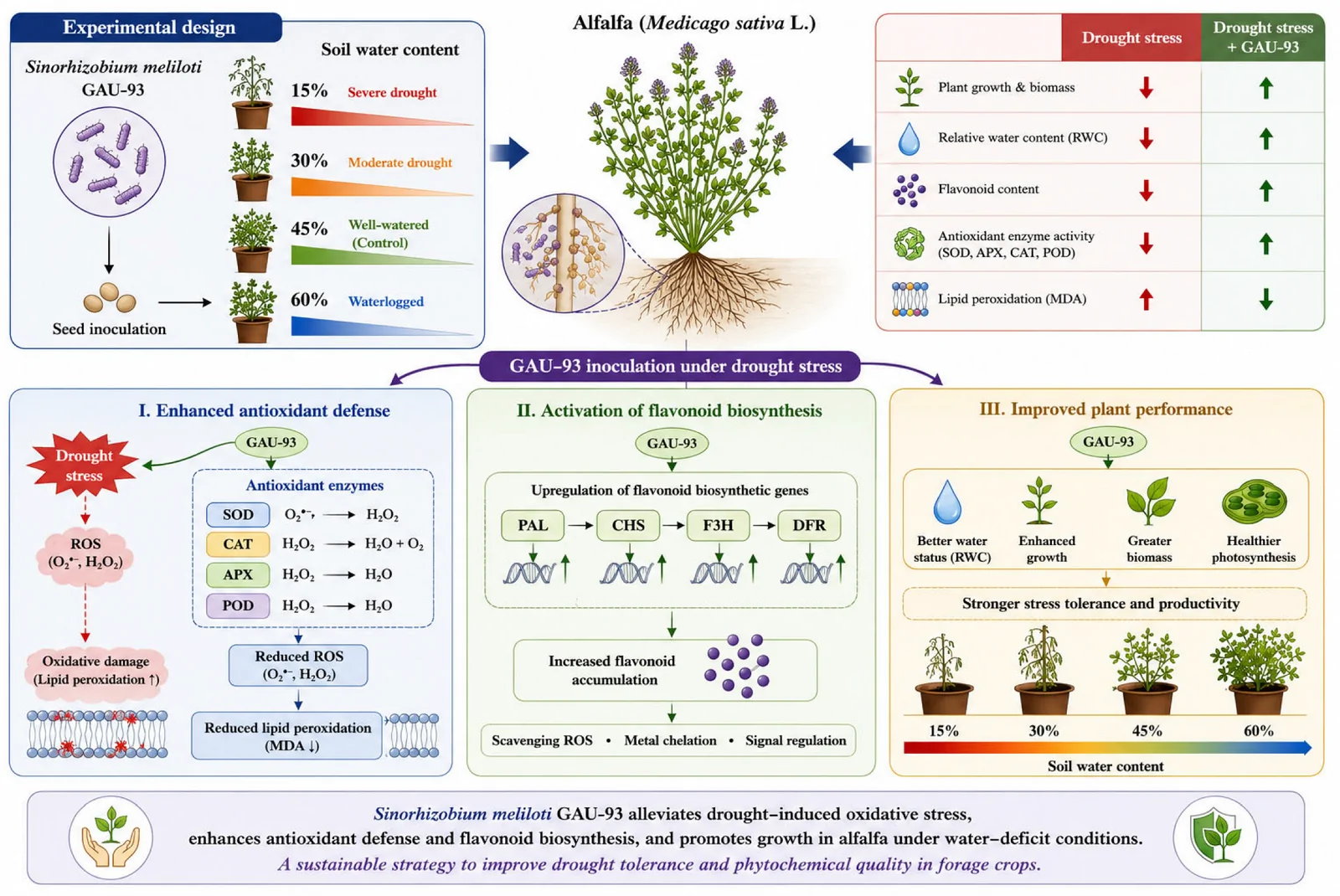
Schematic model illustrating a possible mechanism by which PGPR strain GAU-93 may contribute to drought response in alfalfa. GAU-93 was associated with changes in ROS and oxidative markers, including H_2_O_2_ and MDA, together with changes in antioxidant enzyme activities and flavonoid-related gene expression and enzyme activity. This model summarizes the observed trends without implying direct causality.

Abiotic stressors such as drought can limit plant growth and productivity. In this study, GAU-93 inoculation was associated with lower MDA and H_2_O_2_ accumulation, lower ROS staining in some assays, and higher activities of several antioxidant enzymes. GAU-93 was also associated with higher expression of several flavonoid-related genes and higher flavonoid-related enzyme activity at several SWC levels. These findings suggest a possible association between GAU-93 inoculation and changes in antioxidant and secondary metabolic pathways; however, the involvement of these pathways in improving drought responses remains inferential and requires further experimental validation. The proposed model summarizing these associations is shown in Figure 7.

## Data Availability

The data supporting the findings of this study are available within the article and its Supplementary Materials.

## Supplementary Materials

The following supporting information can be downloaded at: https://www.mdpi.com/article/10.3390/antiox15081040/s1, Table S1, Primer sequences used for qRT-PCR analysis.
