# Supplementary material for: Sinorhizobium meliloti GAU-93 Inoculation Is Associated with Reduced Drought-Induced Oxidative Damage and Enhanced Flavonoid Accumulation and Antioxidant Capacity in Alfalfa
Source: Antioxidants (Basel). 2026 Aug 21;15(8):1040. doi: 10.3390/antiox15081040 (PMC13509790; doi:10.3390/antiox15081040)
Supplement: Supplementary file 1 [file antioxidants-15-01040-s001.zip › antioxidants-4454295-supplementary.pdf]

Table S1 Information of primer sequences of real-time RT-PCR

Gene name	Forward primer(5’-3’)	Reverse primer(5’-3’)
MsPAL	GCAACTCATAGTACCGGCGT'	TGAAGGGCACCACCTTGTTT
MsCHS	ATGAGCGCCACGAGGGAA	GCGCAAGACAACAGTCTCAAT
MsF3H	TTAATTTGCGCGTGGTGCAT	TGTTGCTACGGATAAACGGCT
MsDFR	CCAGATCAATTGGAGCTTGTGAG	CTTTAACAAATTCAGGAAGAAGCCC
MsNOD-1	TGTCTGTGCTCAGTTCAGGTTGC	GCTCCATGCCTCTCAACACCTTC
MsNOD-2	TCACAGCCAATCAAGCCATCATCG	TGTGGACCGTTGTTGTTGGAAGC
MsActin	GACAATGGAACTGGAATGG	CAATACCGTGCTCAATGG
